# Effect of Micro-Structure and Mechanical Properties on 309L Austenitic Stainless Steel Deposition by Cold Metal Transfer Wire-Arc Additive Manufacturing

**DOI:** 10.3390/ma19204275

**Published:** 2026-10-10

**Authors:** Guosheng Fei, Zuofa Liu, Zhongli Li, Xiaoci Chen, Shangbin Zhang, Yingyan Yu, Jiansheng Zhang

**Affiliations:** 1 College of Materials Engineering, Sichuan Polytechnic University, Deyang 618000, China; 2 Chongqing Key Laboratory of Advanced Mold Intelligent Manufacturing, College of Materials Science and Engineering, Chongqing University, Chongqing 400044, China; 3 Chongqing Jiepin Technology Co., Ltd., Chongqing 401329, China

**Keywords:** cold metal transfer (CMT), wire-arc additive manufacturing (WAAM), 309L austenitic stainless steel, process parameters, microstructure, mechanical properties

## Abstract

Wire-arc additive manufacturing based on cold metal transfer (CMT-WAAM) is a high-precision forming technology suitable for thin-wall stainless steel parts, yet the unclear correlations between linear energy density, forming stability, microstructure evolution and mechanical anisotropy restrict the precision forming and performance control of 309L austenitic stainless steel components. Aiming at the scientific problems of end collapse defects and mechanical performance anisotropy in multi-layer thin-wall WAAM forming of 309L stainless steel, this study systematically explores the regulation mechanism of process parameters and deposition path on forming quality, microstructure characteristics and mechanical properties. The optimal single-track forming process window (wire-feeding speed of 5 m/min, welding speed of 3 mm/s) for 309L deposits is determined using single-track single-layer experiments. Compared with unidirectional deposition, the reciprocating deposition path effectively balances inter-layer temperature field distribution, solves the end collapse problem of multi-layer thin-wall structures, and controls the collapse height within 5 mm after 35 layers of continuous deposition. The variation of linear energy density is the core factor dominating microstructure evolution and performance difference: with an increase in linear energy density, the retained delta ferrite in the matrix transforms from a discrete vermicular to continuous skeletal structure, and austenite grains are significantly coarsened. In terms of mechanical properties, the longitudinal tensile performance of the deposits is stable and less affected by heat input, while the improvement of inter-layer metallurgical bonding quality significantly improves the transverse tensile strength and elongation, effectively weakening the mechanical anisotropy of the formed parts. Meanwhile, grain coarsening and the reduction of delta ferrite content led to a gradual decrease in the average Vickers hardness of the deposits. This study reveals the inherent process–structure–performance correlations of CMT-WAAM formed 309L austenitic stainless steel, clarifies the key mechanism for suppressing forming defects and regulating anisotropic performance, and provides theoretical and technical support for the high-quality forming and engineering application of stainless steel WAAM manufacturing and component repair.

## 1. Introduction

Wire-arc additive manufacturing (WAAM) adopts electric arc as the welding/metal-transfer process and realizes near-net-shape forming of components by layer-by-layer deposition of metal wires. Compared with laser-based and electron beam-based additive manufacturing technologies, WAAM possesses outstanding advantages of high material utilization rate, short manufacturing cycle, low equipment cost and high deposition efficiency up to several kilograms per hour [1,2]. These merits make WAAM particularly suitable for the rapid fabrication and repair of large and complex metallic components, and it has been widely applied in aerospace, marine engineering and energy equipment fields in recent years [3,4].

Cold metal transfer (CMT) is an advanced short-circuit welding technology that realizes droplet transfer using wire reciprocating motion, enabling precise low-heat-input deposition with low spatter, minimal deformation and excellent process stability [5,6]. Widely applied in nuclear and hydraulic equipment owing to superior corrosion and high-temperature resistance, 309L austenitic stainless steel is highly heat-sensitive, and its thin walls are prone to thermal deformation, grain coarsening and collapse defects during wire-arc additive manufacturing (WAAM) [7,8]. Compared with TIG, plasma and laser-arc welding, CMT avoids the excessive heat accumulation of conventional arc processes and the high cost and poor adaptability of laser-arc hybrid technology. Its intermittent heat input stabilizes the molten pool and suppresses forming defects and microstructure deterioration, making CMT optimal for high-quality WAAM of 309L thin-wall structures. Process parameters critically govern its molten pool behavior, microstructure evolution and final mechanical properties [9,10].

Extensive investigations have been conducted on the WAAM of austenitic stainless steels in recent years. Previous studies have demonstrated that heat input level exerts a significant influence on the forming accuracy and microstructure-mechanical properties of WAAM components [11,12]. Chen et al. [13] examined the microstructure and mechanical anisotropy of wire-arc additively manufactured austenitic stainless steels (304, 308L, 316L) using EBSD and 60 tensile tests across five orientations and two surface conditions, with DIC employed to capture stress–strain responses. Zheng et al. [14] studied the cyclic behavior of wire-arc additively manufactured stainless-steel shear plates for seismic energy dissipation, showing that WAAM specimens matched hot-rolled counterparts in energy absorption; topology-optimized plates achieved superior stiffness and dissipation, and a validated hysteretic model was established. Kannan et al. [15] fabricated 321 steel by wire-arc additive manufacturing with equiaxed, columnar and elongated grains (3.6–5.9 FN ferrite), achieving higher hardness and tensile strength than conventional 321 steel; although properties declined from room temperature to 900 °C, the WAAM alloy retained 135 ± 7 MPa versus 108 ± 6 MPa, with serrated yielding at 600 °C due to dynamic strain aging. Tanwar et al. [16] produced 316L-309 duplex thin-walled parts by wire-arc additive manufacturing and heat-treated them at 400, 950 and 1200 °C for 2 h. As-built samples contained acicular ferrite in a finer austenite matrix, and 400 °C induced negligible changes, whereas 950 and 1200 °C markedly reduced strength. At 1200 °C, elongation nearly doubled, while strength fell by 14.3%, and hardness dropped 25–35 HV. Rahimi et al. [17] characterized 310 austenitic stainless steel made by cold-metal-transfer wire-arc additive manufacturing, finding epitaxially grown columnar grains with interdendritic and grain-boundary ferrite—unlike the fully austenitic, fine grains of hot-rolled AISI 310; dendrite spacing varied with cooling rate and inter-layer remelting, and the anisotropic microstructure governed direction-dependent tensile and Charpy impact properties.

Despite these research advances, most existing studies on stainless steel WAAM focus on 304 and 316L alloys. For 309L stainless steel, which is widely used for dissimilar steel welding and component repair [18,19], relevant research is still limited. Although several investigations have been carried out on the solidification mode and ferrite morphology evolution of CMT-deposited 309L stainless steel, the intrinsic effects under the coupling effect of different key process parameters still require further in-depth exploration. In addition, the collapse mechanism during thin-wall fabrication and the effect of deposition path strategy still need further investigation to guide practical production.

In view of the above research gaps, this work takes 309L austenitic stainless steel as the research object. Single-track single-layer deposition experiments are first performed to optimize the process parameter window by evaluating bead geometry and surface morphology. On this basis, single-track multi-layer thin-wall specimens under different process parameter combinations are fabricated. The effects of wire-feeding speed, welding speed and deposition path on the forming characteristics of thin-wall specimens are systematically analyzed, and the evolution of the microstructure and mechanical properties as a function of process parameters is revealed. The underlying mechanisms are discussed based on solidification theory and relevant strengthening mechanisms. The research results can provide a theoretical basis and technical guidance for process regulation and quality control of 309L stainless steel CMT-WAAM.

## 2. Experimental Materials and Methods

### 2.1. Experimental Materials

309L austenitic stainless steel wire with a diameter of 1.2 mm was adopted as the deposition material in this experiment. The chemical composition of the welding wire is listed in Table 1. A 304 stainless steel plate with a thickness of 10 mm was selected as the substrate. Prior to deposition, the substrate surface was ground using SiC abrasive papers to remove oxide scales and contaminants, followed by ultrasonic cleaning in ethanol for 10 min to eliminate grease. 309L stainless steel possesses favorable mechanical properties, including high compressive resistance, superior tensile strength, good ductility, and excellent weldability, as well as outstanding corrosion-resistance performance [20,21].

### 2.2. Experimental Equipment

The additive manufacturing system consists of a Fronius^®^ VR 7000 CMT digital welding power source, a KUKA six-axis industrial robot, a wire-feeding mechanism and a shielding gas system. The welding power source enables precise control over the CMT short-circuit transfer process. The motion trajectory and travel speed of the welding torch are controlled by the robot using offline programming. A mixed gas of 97.5% Ar + 2.5% CO_2_ was employed as the shielding gas, with the gas flow rate set at 20 L/min.

Before deposition, no preheating was applied to the substrate. The substrate was clamped on a water-cooled copper fixture during the whole WAAM process. Inter-layer temperature was monitored by an infrared thermometer, and deposition of the subsequent layer started only when the inter-layer temperature cooled down to 100 °C. No external forced cooling was used except the water-cooled fixture.

### 2.3. Experimental Scheme

#### 2.3.1. Optimization of Single-Track Single-Layer Deposition Process

The control-variable method was adopted to conduct 22 sets of single-track single-layer deposition experiments, where the wire-feeding speed and welding speed ranged from 2–6 m/min and 2–6 mm/s, respectively. The detailed experimental scheme is listed in Table 2. Parameter combinations with favorable forming quality were screened by means of macroscopic morphology observation, measurement of clad width and reinforcement height, and calculation of the reinforcement height coefficient.

The weld reinforcement form factor *φ*, a dimensionless evaluation index, is defined as the ratio of bead width *W* to reinforcement height *H*, with the mathematical expression of *φ* = *W*/*H*. This index can quantitatively characterize the fullness and geometric stability of single-track weld bead formation. A reasonable value of the reinforcement form factor corresponds to a smooth weld cross-section profile and superior forming quality. An excessively large factor tends to produce overly flat weld beads with insufficient cladding thickness, whereas an excessively small factor leads to prominent weld humps, which may readily induce forming defects during subsequent multi-layer deposition [22,23].

The linear energy density (heat input) *E* is calculated according to Equation (1):

(1) *E* = (*I* × *U*)/*v*

where *I* is the welding current (A), *U* is the welding voltage (V), and *v* denotes the welding speed (mm/s). The unit of linear energy density *E* is J/mm. This value represents the total electrical power input without considering the thermal efficiency of the CMT arc process, rather than the effective heat transferred into the workpiece. This physical quantity characterizes the heat input per unit length of the cladding track, and acts as a critical process parameter for regulating heat accumulation, microstructure evolution and geometric forming characteristics during the WAAM process [24,25].

#### 2.3.2. Fabrication of Single-Track Multi-Layer Thin-Wall Specimens

According to the process law derived from single-track single-layer deposition experiments, four sets of representative linear energy density parameters were selected to fabricate single-track multi-layer thin-wall specimens. Wire-feeding speed and welding speed were selected as primary variables, while voltage and current were matched accordingly to realize gradient linear energy density. The parameter combinations were confined within the stable process window obtained from preliminary single-track single-layer cladding tests.

The designed height of the thin-wall specimens was approximately 40 mm, and the inter-layer temperature was controlled at 200 °C, which was monitored in real time using a contact thermometer. A linear deposition path was employed for all specimens, with the welding speed ranging from 2 mm/s to 4 mm/s and the wire-feeding speed varying from 3 m/min to 6 m/min. The complete process parameters of each specimen are summarized in Table 3.

The target building height of 40 mm and corresponding 35 deposited layers were selected to ensure sufficient thermal accumulation characteristics and stable microstructure evolution along the building direction. This height can eliminate the strong thermal disturbance from the substrate in the initial several layers and obtain a relatively steady-state thermal field in the middle and upper regions of the thin wall, which is suitable for investigating the microstructure and mechanical properties under quasi-stable WAAM thermal cycling. Preliminary trials showed that a lower height would keep the whole sample under strong substrate heat influence, while excessively high walls risked geometric distortion and collapse during deposition.

### 2.4. Characterization and Testing Methods

Metallographic specimens were sectioned from the middle region of thin-wall samples. The specimens were ground step-by-step using P80 to P1200 grit papers and further polished with diamond polishing agent. Subsequently, the specimens were etched for 15–20 s with an HCl-to-HNO_3_ ratio of 3:1. The microstructural features of the deposition zone and heat-affected zone were observed using a Leica DM2700M optical microscope.

Plate-shaped tensile specimens were machined along both the parallel (longitudinal) and perpendicular (transverse) directions relative to the deposition direction according to the ISO 6892-1:2019 standard [26]. The gauge length, width, and thickness of tensile specimens were 25 mm, 6 mm and 2 mm, respectively. Room-temperature tensile tests were carried out on a universal material testing machine at a constant strain rate of 1 mm/min. Three parallel specimens were tested for each process condition, and the average value was adopted to reduce experimental scatter.

Vickers hardness measurements were performed using an HV-1000 hardness tester under an applied load of 0.98 N with a dwell time of 15 s. Hardness indentations were made at an interval of 0.5 mm along the deposition direction, and the averaged value of multiple measuring points was regarded as the representative hardness of the specimen.

## 3. Results and Discussion

### 3.1. Forming Characteristics and Process Analysis of Single-Track Single-Layer Deposited Beads

#### 3.1.1. Bead Cross-Sectional Geometry

**Figure 1:**
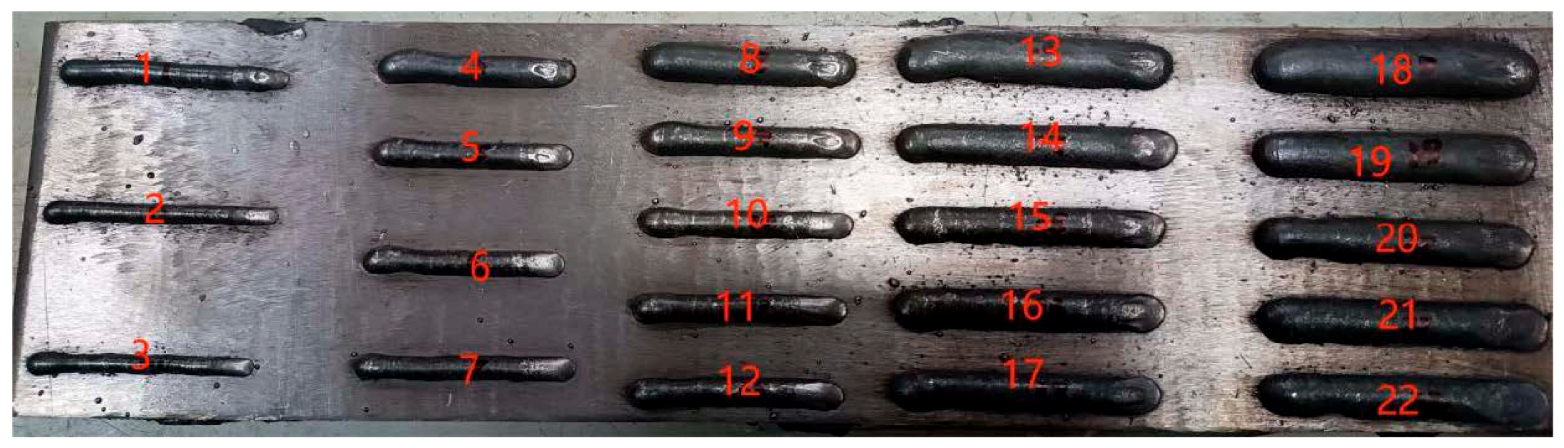
Macro-surface morphology of single-track single-layer deposited beads.

The experimental results of single-track single-layer deposition are presented in Figure 1. After fabrication of the weld beads, the bead width and reinforcement height were quantitatively measured, and the corresponding weld reinforcement form factor was further calculated. The measured geometrical data under various process parameters are summarized in Table 4.

Welding process parameters, such as wire-feeding speed and welding speed, exert a remarkable influence on the forming quality of deposited layers [27,28]. To reveal the underlying mechanism of these critical parameters on bead geometry and identify the optimal process window, the effects of wire-feeding speed and welding speed on the morphological characteristics of deposited layers were investigated in the following section.

#### 3.1.2. Effect of Wire-Feeding Speed on Morphological Features

**Figure 2:**
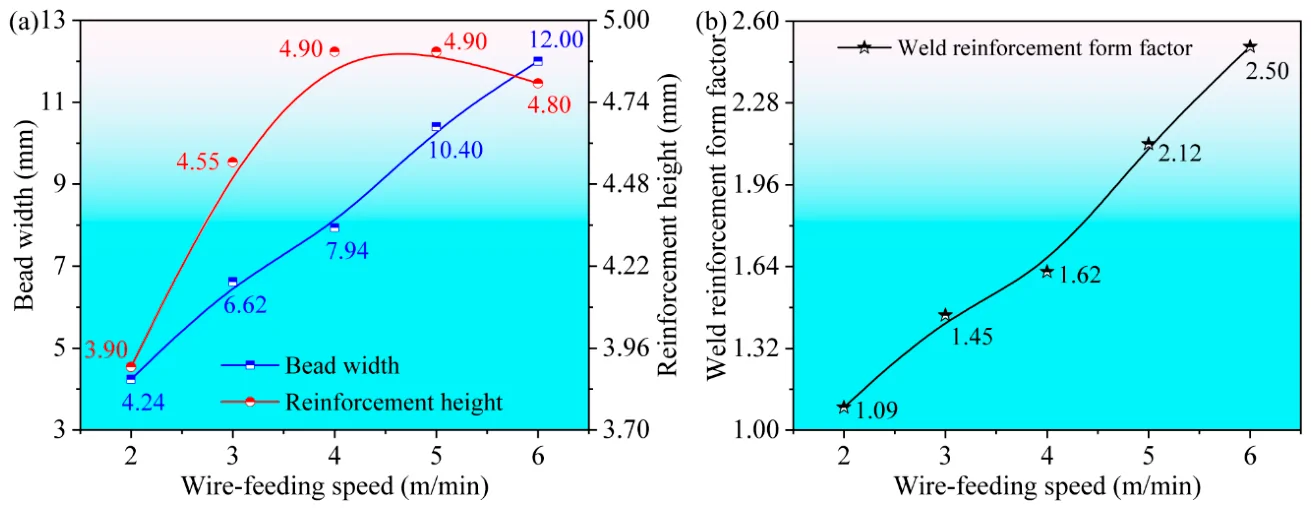
Variations of geometric parameters of single-track single-layer deposited beads with wire-feeding speed: (**a**) bead width and reinforcement height; (**b**) weld reinforcement form factor.

**Figure 3:**
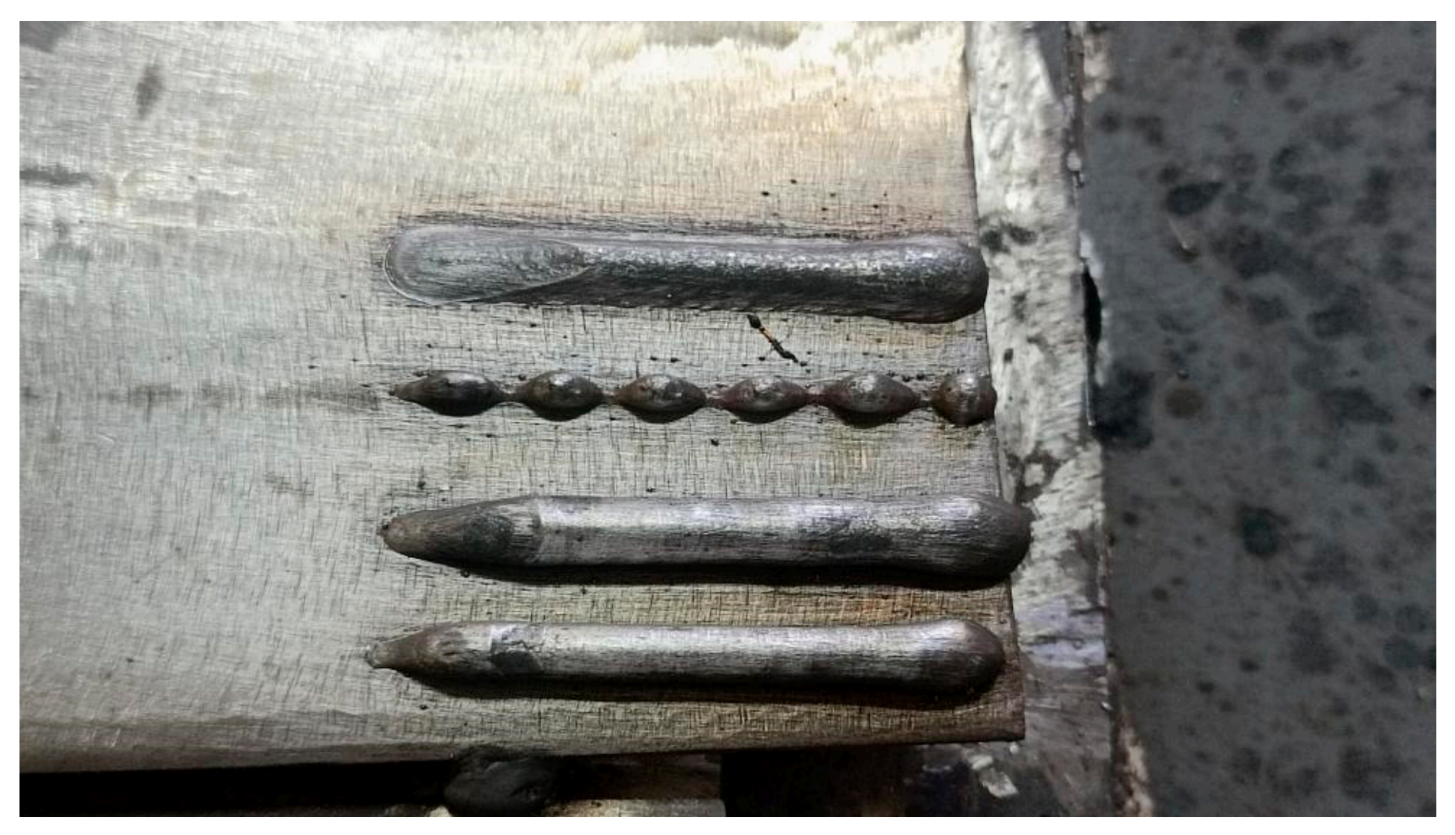
Defective macro-morphology of deposited bead at wire-feeding speed of 2 m/min.

Single-track single-layer deposition experiments were conducted using cold metal transfer (CMT)-based wire-arc additive manufacturing (WAAM) at a constant travel speed of 2 mm/s, with wire-feeding speed set at 2, 3, 4, 5 and 6 m/min, respectively. The geometric characteristics and macroscopic morphology of deposited beads at different wire-feeding speeds were systematically characterized, as presented in Figure 2 and Figure 3.

Figure 2 illustrates the variation trends of bead geometric parameters with increasing wire-feeding speed. As shown in Figure 2a, bead width increased monotonically from 4.24 mm to 12.00 mm as wire-feeding speed rose from 2 m/min to 6 m/min, showing an approximately linear growth trend. The reinforcement height first increased from 3.90 mm to a peak of 4.90 mm at a wire-feeding speed of 4–5 m/min, and then slightly decreased to 4.80 mm at 6 m/min. Correspondingly, the weld reinforcement form factor rose continuously from 1.09 to 2.50 with wire-feeding speed increasing from 2 to 6 m/min (Figure 2b), indicating that higher wire-feeding speed promoted lateral spreading of the molten pool and led to a wider and flatter bead profile. Each geometric value is the average of three repeated measurements at different positions along the weld bead.

In the CMT additive manufacturing process, the power source automatically adjusts welding current and voltage to match the preset wire-feeding speed. The rise in wire-feeding speed leads to a synchronous increase in arc power and heat input, which enhances filler wire melting and improves molten metal fluidity [29,30]. The increased metal deposition volume per unit length directly enlarges the overall bead dimensions; meanwhile, elevated heat input improves wettability between the molten pool and substrate, facilitating lateral flow of molten metal. This is the main reason why bead width grows more significantly than reinforcement height at higher wire-feeding speed.

Along the length of a single deposited bead, three morphological zones with distinct features can be observed. At the arc initiation zone, arc current and voltage rise gradually while wire-feeding speed reaches the preset value rapidly, resulting in insufficient initial heat input and poor molten metal fluidity. This condition usually causes a locally convex bead profile and increases the risk of defects such as undercut. In the middle stable deposition zone, arc parameters, wire-feeding speed and travel speed reach a balanced state with stable heat input. The deposited bead in this zone exhibits a smooth and uniform surface with distinct fish-scale ripples, representing the main weld body with sound forming quality. At the arc termination zone, the reduced travel speed breaks the balance between wire feeding and welding travel, causing excessive heat input and expanded molten pool area, which results in a rougher bead surface and more severe spatter [31,32].

When wire-feeding speed was maintained at a low level (2–3 m/min), the overall forming quality of deposited beads deteriorated dramatically, and discontinuous bead formation even occurred, as shown in Figure 3. Insufficient heat input under low wire-feeding speed could not sustain a stable continuous molten pool, and poor molten metal fluidity led to discontinuous deposition and balling defects, which severely degrade the deposition quality.

As wire-feeding speed increased to the range of 4–5 m/min, heat input and metal deposition reached an optimal balance, and the deposited beads presented no obvious defects with favorable surface finish. When wire-feeding speed further increased to 6 m/min, the wire-feeding volume per unit length rose remarkably, causing a substantial increase in bead width and a slight reduction in reinforcement height. The higher heat input also strengthened the preheating effect on the substrate, which further improved molten pool fluidity. The enhanced flowability promoted molten metal filling in the bead and reduced the occurrence probability of porosity and inclusions to some extent. Based on repeated experimental measurements and morphological evaluation, the optimal bead-forming quality was achieved at a wire-feeding speed of 5 m/min, which was selected as the preferred parameter for subsequent multi-layer deposition experiments.

#### 3.1.3. Effect of Welding Speed on Morphological Features

Single-track single-layer deposition experiments were conducted at a constant wire-feeding speed of 5 m/min, with welding speed set at 2, 3, 4, 5 and 6 mm/s, respectively. Within the tested parameter range, all deposited beads presented generally favorable forming quality without obvious welding defects, as demonstrated in Figure 1.

**Figure 4:**
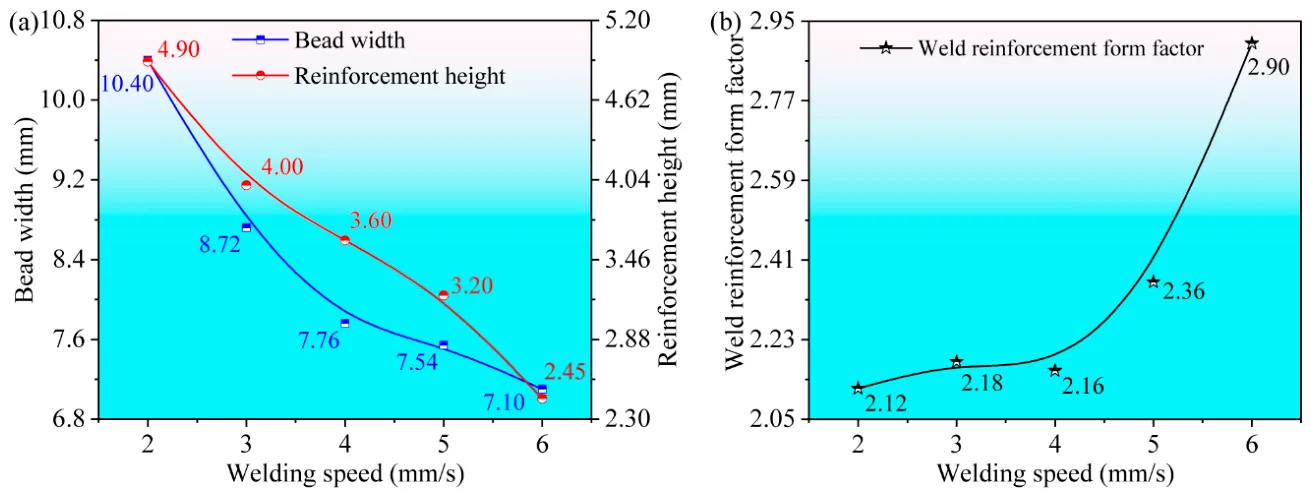
Variations of geometric parameters of single-track single-layer deposited beads with welding speed: (**a**) bead width and reinforcement height; (**b**) weld reinforcement form factor.

Figure 4 illustrates the variation trends of bead geometric parameters with increasing welding speed. As shown in Figure 4a, both bead width and reinforcement height decreased continuously with the rise in welding speed. Bead width declined from 10.40 mm at 2 mm/s to 7.10 mm at 6 mm/s, and reinforcement height was reduced correspondingly from 4.90 mm to 2.45 mm. The decreasing amplitude was more remarkable at lower welding speeds and became gradual as welding speed further increased. When wire-feeding speed remained unchanged, an increase in welding speed led to a reduction in heat input per unit length of the deposited bead, which weakened the fluidity of molten metal in the molten pool [33,34]. Meanwhile, higher welding speed also decreased the volume of filler metal deposited per unit travel distance. The combined action of these two factors resulted in the continuous shrinkage of bead width and reinforcement height.

As presented in Figure 4b, the weld reinforcement form factor (φ), defined as the ratio of bead width to reinforcement height, exhibited an overall increasing trend with rising welding speed. The value grew from 2.12 to 2.90 as welding speed increased from 2 mm/s to 6 mm/s, with slight fluctuations observed at intermediate speed levels. This trend suggested that reinforcement height decreased at a faster rate than bead width under higher welding speeds, leading to a relatively wider and flatter bead profile.

In terms of surface morphology, the bead fabricated at a welding speed of 2 mm/s showed an uneven and rough surface with obscure fish-scale ripples, irregular contour and non-uniform bulges. Such poor forming uniformity makes this parameter inappropriate for additive manufacturing processes. By comparison, beads produced at welding speeds of 3 mm/s and above possessed much better overall uniformity and surface consistency, and these parameters could be regarded as feasible alternatives for multi-layer deposition.

Based on repeated experimental comparisons, a welding speed of 3 mm/s delivered the most appropriate weld reinforcement form factor. Deposited beads prepared under this parameter showed no detectable welding defects, and the reinforcement height maintained at a stable and moderate level, which was beneficial to guarantee consistent layer thickness in the subsequent multi-layer additive manufacturing process.

### 3.2. Forming Characteristics and Process Analysis of Single-Track Multi-Layer Thin-Wall Specimens

#### 3.2.1. Effect of Deposition Paths on Forming Characteristics of Thin-Wall Specimens

Different from conventional metal inert gas/metal active gas welding, CMT welding features rapid arc ignition and arc extinction, which enables melting of base metal within a short duration. At the arc initiation position of each weld bead, the initial thermal state differs from the stable inter-layer temperature of the previous layer, leading to poor fluidity of molten metal and a larger bead height compared with the middle section of the weld bead [35,36]. The forming quality at both arc initiation and arc termination regions is generally unsatisfactory. At the arc initiation side, low initial inter-layer temperature and insufficient heat input result in poor fluidity and fast solidification of deposited metal, leading to a hemispherical convex profile. At the arc termination side, preheating from subsequent deposition layers elevates the temperature of preceding beads and improves the fluidity of molten metal. Driven by arc force, gravity and impact force of droplet transfer, the molten pool forms a concave shape, which leaves a pit after solidification [37,38]. Such non-uniform forming at bead ends is amplified during multi-layer accumulation, making deposition path strategy a critical factor affecting the dimensional accuracy of thin-wall structures.

**Figure 5:**
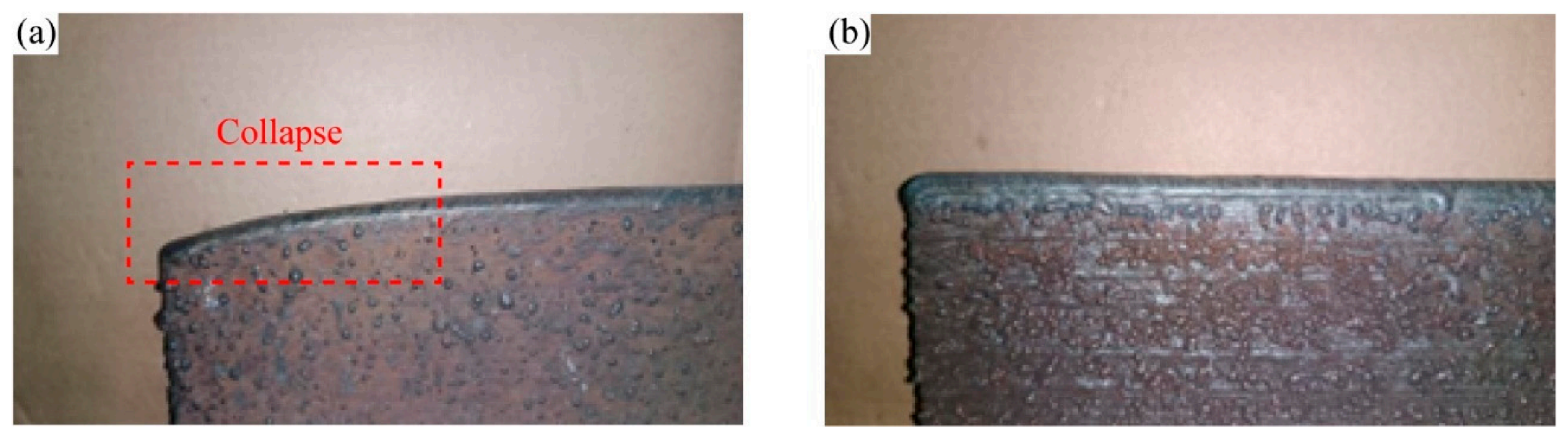
Forming characteristics of samples under different deposition paths: (**a**) unidirectional deposition path; (**b**) reciprocating deposition path.

In this work, two typical deposition paths, namely unidirectional deposition path and reciprocating deposition path, were compared to investigate their influence on the forming quality of thin-wall specimens, as shown in Figure 5. The unidirectional path refers to all deposition layers traveling along the same direction, while the reciprocating path adopts opposite traveling directions for adjacent layers, with the arc termination point of the upper layer serving as the arc initiation point of the lower layer. As can be observed from Figure 5a, the thin-wall sample fabricated with the unidirectional path presents an obvious collapse defect at the end region, accompanied by irregular sidewall profile and poor top flatness. In contrast, the sample prepared with the reciprocating path exhibits uniformly flat sidewalls and a continuous straight top contour, as presented in Figure 5b, demonstrating significantly improved overall forming quality.

The discrepancy in forming quality mainly arises from the differences in heat input distribution and inter-layer temperature evolution under the two path strategies. For the unidirectional deposition mode, the arc initiation and arc termination positions are fixed at the same ends for all layers, causing a persistent imbalance of heat accumulation at both ends. The arc initiation end repeatedly undergoes low-temperature arc starting, which leads to insufficient fluidity of deposited metal and gradually increased accumulation height layer by layer. The arc termination end suffers from continuous arc heating and excessively high inter-layer temperature, which greatly enhances molten metal fluidity. The overheated molten metal flows downward under gravity, and the underlying solidified layer cannot provide sufficient support for the flowing metal. This process results in a narrowed upper section and widened lower section of the thin wall, and eventually induces the collapse defect [39,40]. For the reciprocating deposition mode, arc initiation and arc termination alternate at both ends of the deposit, which effectively balances the inter-layer temperature distribution, avoids excessive local heat accumulation, and improves the solidification uniformity of the molten pool.

Apart from thermal distribution, residual stress also plays an important role in the formation of collapse. During multi-layer additive manufacturing, residual stress inside the weld beads and at inter-layer interfaces accumulates continuously with increasing layer number, and does not dissipate at the bottom as the deposit height increases. When the accumulated residual stress exceeds the yield strength of the material, plastic deformation occurs at the weak region, which further aggravates the end collapse [41,42]. In addition, the collapse behavior is also associated with other factors, including heat input level, fluidity of deposited metal and cooling rate. Excessively high heat input improves molten metal fluidity and aggravates gravity-driven downward flow, which promotes the occurrence of collapse. In contrast, excessively fast cooling rate restricts the release of internal stress and also increases the risk of collapse.

The reciprocating deposition path can effectively suppress the end collapse defect and improve the forming consistency of thin-wall structures. Under the optimized parameters with a wire-feeding speed of 5 m/min and a welding speed of 3 mm/s, the collapse height at the end was controlled within 5 mm after 35 layers of deposition, which met the precision requirement for subsequent experiments. Accordingly, the reciprocating deposition path was adopted in all subsequent multi-track multi-layer deposition experiments.

#### 3.2.2. Effect of Wire-Feeding Speed on Forming Characteristics of Thin-Wall Deposits

Wire-feeding speed serves as a critical process parameter that dominates the forming quality of thin-wall structures in multi-layer wire-arc additive manufacturing. It determines the volume of filler metal deposited per unit length, and thus directly regulates the geometric dimensions of individual deposition beads and the macroscopic morphological characteristics of the overall structure [43,44].

At low wire-feeding speeds, the amount of molten metal deposited per unit travel distance is limited, leading to reduced bead width and reinforcement height. The insufficient layer thickness weakens the vertical structural support of the thin wall during continuous stacking, making the deposit susceptible to collapse and dimensional deformation. Furthermore, inadequate metal feeding under low wire-feeding speeds may cause discontinuous bead formation and uneven sidewall profiles, further compromising the geometrical accuracy of the fabricated component [45,46].

When wire-feeding speed is excessively high, the overall dimensions of single beads increase, yet various forming flaws and internal defects emerge simultaneously. Despite the rise in total heat input accompanying higher wire-feeding speed, the energy density per unit volume of molten metal decreases, which impairs the fluidity of deposited metal and increases the probability of internal defects, including lack of fusion and slag inclusions. Meanwhile, the enlarged volume of deposited metal and elevated total heat input slow down the solidification and cooling rate of the molten pool, which may induce greater residual stress within the deposit and adversely affect the overall mechanical properties of the thin-wall structure.

**Figure 6:**
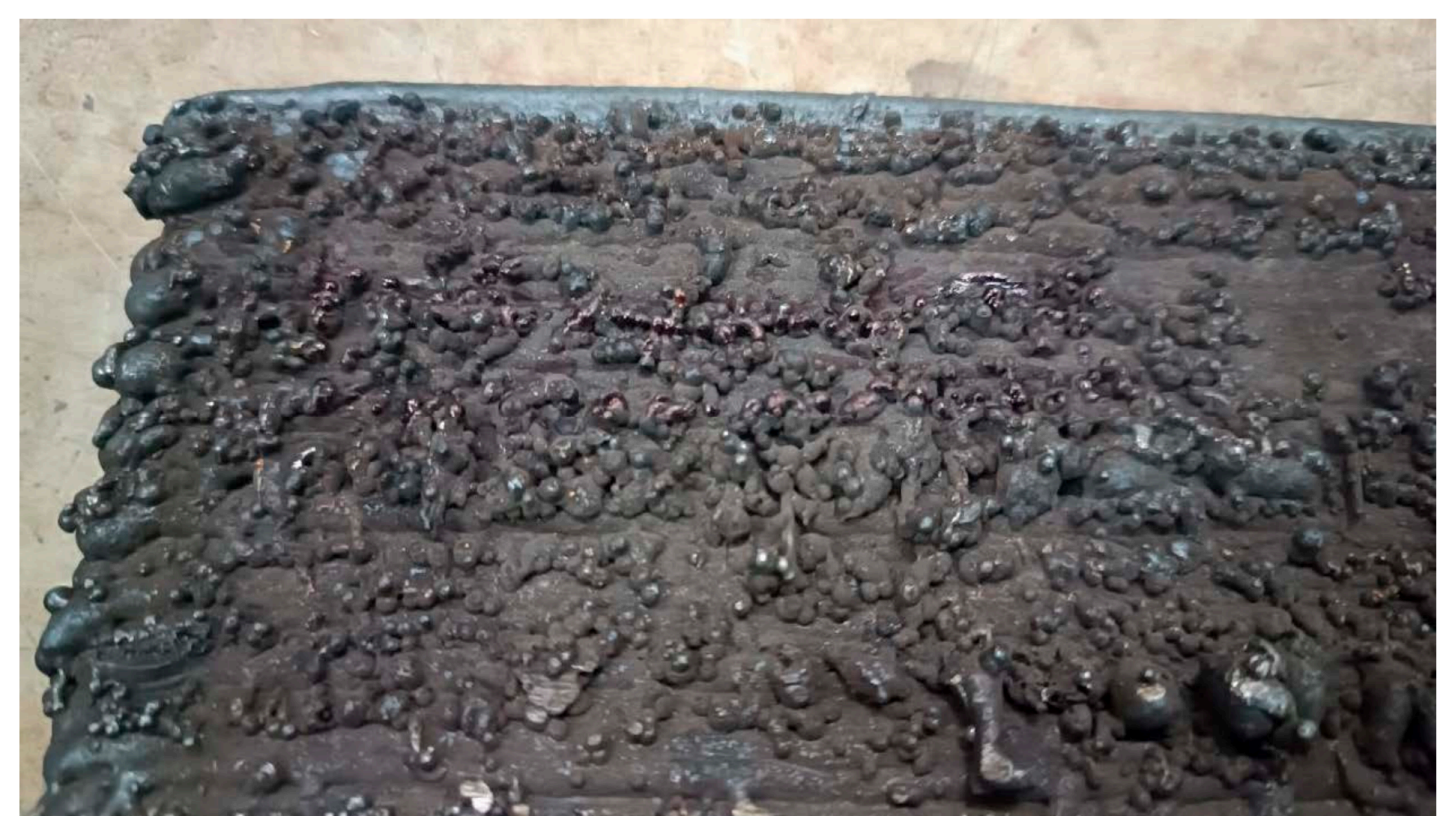
Morphology of flow-down defects of deposited metal on the as-built thin-wall sample.

In addition to internal quality issues, mismatched wire-feeding speed also induces typical surface forming defects on the sidewall of thin-wall deposits, as presented in Figure 6. As the deposition height increases gradually, continuous layer-by-layer heating causes significant heat accumulation inside the thin-wall deposits. The accumulated thermal energy raises the temperature of previously solidified layers and reduces their mechanical support capacity for the newly formed molten pool. With excessive molten metal supply and the action of gravity, part of the molten metal cannot be fully retained by the underlying bead and flows downward along the sidewall surface. Upon solidification, these downward-flowing metal fractions form discrete metallic droplets and irregular bulges of varying sizes on the sidewall. Such flow-down defects degrade both the dimensional precision and surface finish of the as-built thin wall, thereby increasing the machining allowance and processing complexity in subsequent finishing operations.

These findings demonstrate that a properly matched wire-feeding speed is indispensable for balancing the metal deposition rate, heat input level, and molten pool stability, which serves as the basis for fabricating thin-wall structures with uniform geometry, favorable sidewall morphology and suppressed internal defects. Combined with the single-layer parameter optimization results in the preceding section, a wire-feeding speed of 5 m/min is confirmed to be appropriate for multi-layer thin-wall structures. This parameter, in combination with the reciprocating deposition path, was confirmed to achieve appropriately stable forming quality and effectively avoided severe flow-down defects on the sidewall.

#### 3.2.3. Effect of Welding Speed on Forming Characteristics of Thin-Wall Deposits

Welding speed exerts a remarkable effect on the geometric dimensions and macroscopic forming quality of multi-layer thin-wall deposits [47]. As welding speed increases, both the overall wall width and average layer height of the fabricated components present an obvious downward trend. When thin-wall deposits are stacked at a high welding speed of 4 mm/s, the number of deposition layers increases for a given target height, but the sidewall morphology degrades notably. The inter-layer boundaries on the sidewall become indistinct in local areas, and local collapse defects may even occur in some regions.

Such forming deterioration primarily arises from the insufficient interaction time between the arc, molten pool and base material at elevated travel speeds. When welding speed is excessively high, the deposited metal cannot fully melt and spread laterally within the shortened arc action duration, resulting in reduced bead width and reinforcement height as well as insufficient inter-layer bonding between adjacent deposition beads. Furthermore, the accelerated solidification process under high welding speed restricts the effective release of residual stress accumulated during continuous layer-by-layer deposition, which further intensifies the risk of structural collapse and dimensional deformation of the thin-wall component.

**Figure 7:**
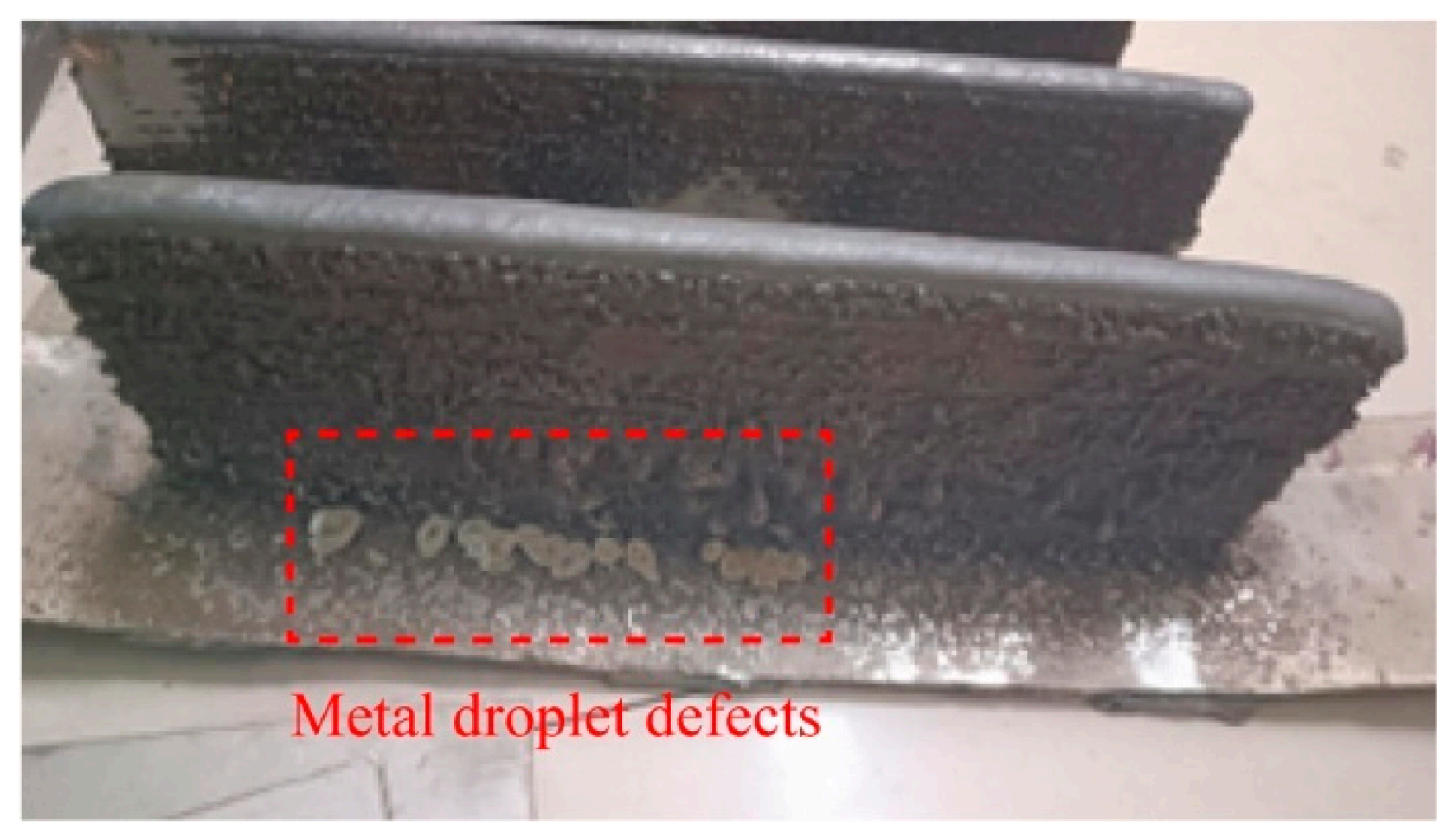
Macro-morphology of metal droplet defects on thin-wall deposits.

In addition to internal bonding performance and structural stability, improper welding speed also causes characteristic surface defects on the sidewalls of thin-wall structures. As illustrated in Figure 7, abundant scattered metal droplet defects are observed on the sidewall and bottom area of the as-built thin wall. These defects are formed by unstable droplet transfer and arc spatter under mismatched process conditions. At excessively high welding speeds, the dynamic equilibrium of the molten pool is broken, and partially melted metal droplets splash out of the deposition zone and adhere to the sidewall surface before complete solidification. The existence of these metal droplet defects reduces both the dimensional precision and surface quality of the thin-wall structure, and raises the difficulty and material removal allowance of subsequent finishing operations.

Based on the experimental results for the wire-feeding speed analyzed in the preceding section, it is concluded that desirable forming quality of thin-wall structures relies heavily on the proper matching between wire-feeding speed and welding speed. When the two process parameters are well coordinated, stable molten pool state, sufficient metal filling and sound inter-layer bonding can be achieved, leading to a uniform geometric profile and favorable macroscopic appearance of the deposited thin wall.

### 3.3. Effect of Linear Energy Density on Microstructure and Mechanical Properties of Thin-Wall Deposits

As described in the previous section, variations in wire-feeding speed and welding speed exert pronounced influences on the macroscopic forming quality of deposited beads. In practical engineering applications, both satisfactory surface morphology and excellent mechanical properties of as-fabricated components are critical performance requirements. As a core parameter in the welding process, linear heat input directly governs the heat accumulation and cooling rate within the weld zone, and further affects grain size evolution and the final mechanical performance of the deposited material [48,49]. This section investigates the microstructure and mechanical properties of different regions in 309L stainless steel deposits fabricated by WAAM under various linear heat input conditions, with all other process parameters maintained constant. The systematic analysis of experimental data can lay a theoretical foundation for subsequent multi-track multi-layer deposition experiments and additive manufacturing repair of hydraulic turbine components.

#### 3.3.1. Effect of Linear Energy Density on Microstructure of Thin-Wall Structures

**Figure 8:**
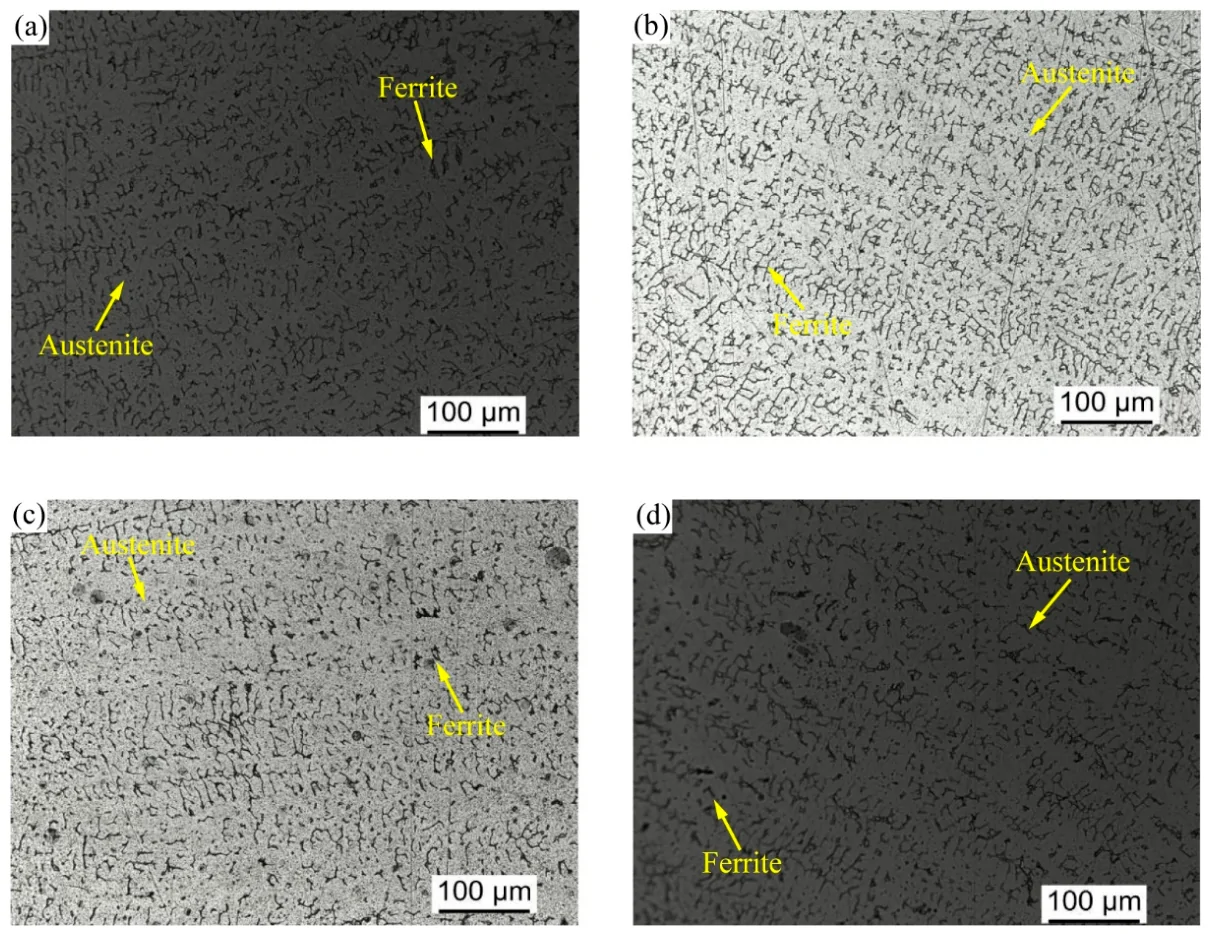
Microstructure of thin-wall deposits under different linear energy densities: (**a**) 348.40 J/mm; (**b**) 481.80 J/mm; (**c**) 642.40 J/mm; (**d**) 824.47 J/mm.

To investigate the influence of linear energy density on the microstructural characteristics of thin-wall structures, metallographic observations were carried out on specimens fabricated under different heat input conditions. The typical microstructures of thin-wall deposits prepared under four different linear energy densities are shown in Figure 8, with the inter-layer temperature maintained at 200 °C during the entire deposition process. It can be seen that the retained delta ferrite dispersed in the austenite matrix presents two typical morphologies: vermicular and skeletal. The formation and evolution of ferrite are closely related to the austenite transformation process: the nucleation and growth of austenite consume the primary delta ferrite formed during solidification, and this phase transformation process is gradually inhibited as the temperature decreases during cooling.

For specimens fabricated under relatively low linear energy densities (348.40 J/mm and 481.80 J/mm, corresponding to Figure 8a and Figure 8b, respectively), the limited heat input shortens the duration available for austenite transformation. As a consequence, a larger amount of delta ferrite is retained in the matrix after cooling to room temperature, and the ferrite mainly exhibits a discrete vermicular morphology.

In comparison, specimens produced under higher linear energy densities (642.40 J/mm and 824.47 J/mm, shown in Figure 8c,d) possess a larger austenite grain size and more distinct grain boundaries, which gradually connect into continuous networks. This phenomenon is attributed to the enhanced grain growth driven by the higher initial heat input. A higher heat input extends the overall high-temperature residence time and promotes austenite grain coarsening. Nevertheless, within the critical temperature interval for δ→γ phase transformation, the local cooling rate still remains relatively high for the WAAM thin-wall deposits. Although high heat input slows the overall cooling process, the thin-wall geometry enables rapid heat loss once the temperature drops into the δ/γ transformation range. The shortened effective time available for δ→γ transformation within this transformation window restrains the full consumption of ferrite. As a result, the retained delta ferrite finally forms a continuous skeletal morphology distributed along the austenite grain boundaries.

#### 3.3.2. Effect of Linear Energy Density on Mechanical Properties of Thin-Wall Deposits

To evaluate the influence of linear energy density on the mechanical performance of as-fabricated thin-wall deposits, room-temperature tensile tests were conducted on specimens prepared under four different heat input conditions, with the inter-layer temperature controlled at 200 °C throughout the deposition process. Considering the inherent microstructural anisotropy of additively manufactured components, tensile specimens were machined along both longitudinal and transverse directions to clarify the directional dependence of mechanical properties. Tensile tests under each linear energy density condition were repeated on three independent specimens.

**Figure 9:**
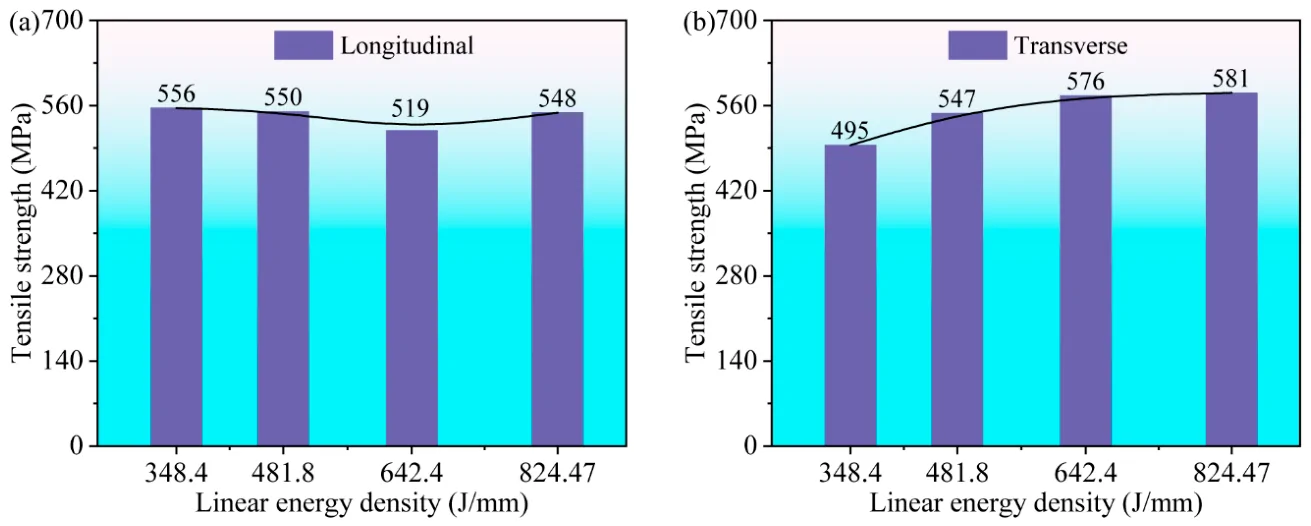
Tensile strength of thin-wall deposits under different linear energy densities: (**a**) longitudinal sampling direction; (**b**) transverse sampling direction.

Figure 9 shows the tensile strength of thin-wall deposits fabricated at different linear energy densities. For specimens tested along the longitudinal direction, the tensile strength exhibits a relatively slight fluctuation with varying heat input, with all values maintaining around 550 MPa. Specifically, the tensile strength reaches 556 MPa at 348.40 J/mm and 550 MPa at 481.80 J/mm, decreases to a minimum of 519 MPa at 642.40 J/mm, and then recovers to 548 MPa at 824.47 J/mm. In contrast, the transverse tensile strength presents a continuous increasing trend with rising linear energy density, growing steadily from 495 MPa at 348.40 J/mm to 581 MPa at 824.47 J/mm. It is noteworthy that at low linear energy densities, the longitudinal tensile strength is obviously higher than the transverse value, while this directional difference gradually narrows as heat input increases.

**Figure 10:**
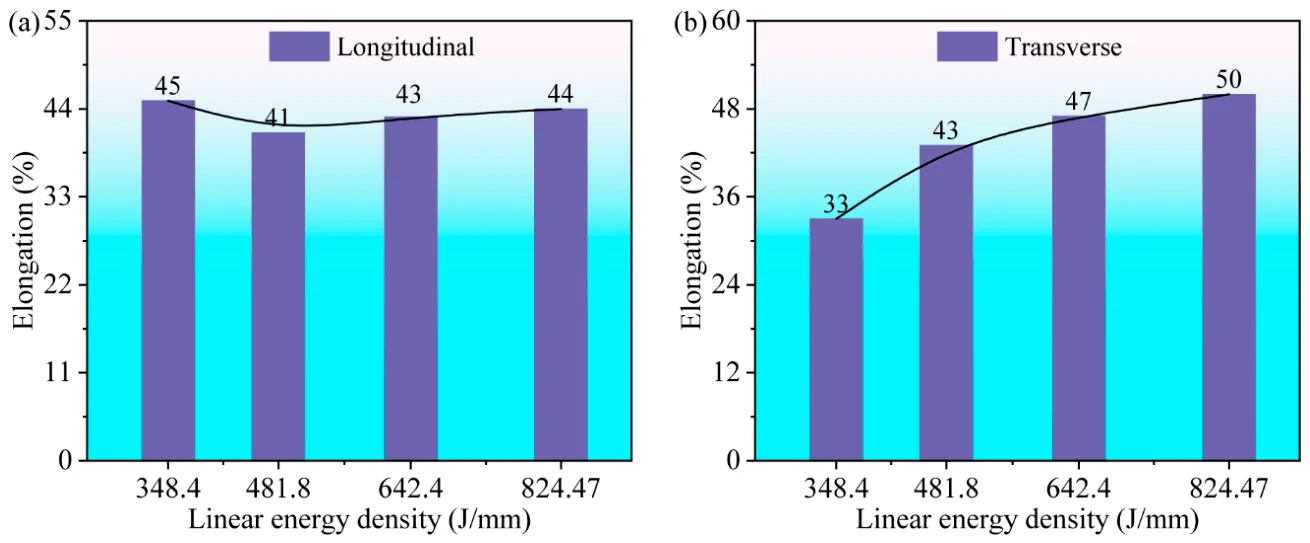
Elongation of thin-wall deposits under different linear energy densities: (**a**) longitudinal sampling direction; (**b**) transverse sampling direction.

The variation of elongation with linear energy density is illustrated in Figure 10. Consistent with the tensile strength trend, the longitudinal elongation remains stable in the range of 41% to 45% across all heat input conditions, showing negligible dependence on linear energy density. For the transverse direction, however, the elongation increases remarkably with elevated heat input, rising from 33% at 348.40 J/mm to 50% at 824.47 J/mm. This result indicates that higher linear energy density can effectively improve the plastic deformability of the deposit in the transverse direction.

The observed mechanical anisotropy and parameter-dependent performance can be attributed to the differences in microstructural characteristics and inter-layer bonding quality between the two sampling directions. For longitudinal specimens loaded parallel to the deposition direction, the loading path mainly passes through the interior of single deposition beads, where the microstructure is relatively homogeneous with fewer inter-layer interfaces. As a result, the longitudinal mechanical properties are less affected by heat input variation and maintain good stability.

For transverse specimens loaded perpendicular to the deposition direction, the tensile path crosses multiple deposition layers and inter-layer bonding interfaces. During multi-layer deposition, each subsequent layer causes partial remelting of the previous layer, forming a remelting zone at the inter-layer boundary. The austenite grains in the remelting zone become relatively coarser under repeated thermal cycling, making the inter-layer interface a mechanically weak region. Under low heat input conditions, insufficient inter-layer remelting may lead to poor metallurgical bonding at the interfaces, which further deteriorates transverse mechanical properties and causes premature fracture at inter-layer junctions. With an increase in linear energy density, enhanced heat input promotes sufficient remelting at the inter-layer regions, improves metallurgical bonding quality, and reduces interface defects. Although higher heat input also induces grain coarsening to some extent, the improvement in inter-layer bonding plays a dominant role, leading to continuous enhancement of both strength and plasticity in the transverse direction. In addition, the variation of residual stress level with heat input also exerts a certain influence on the final mechanical properties of the deposited components.

**Figure 11:**
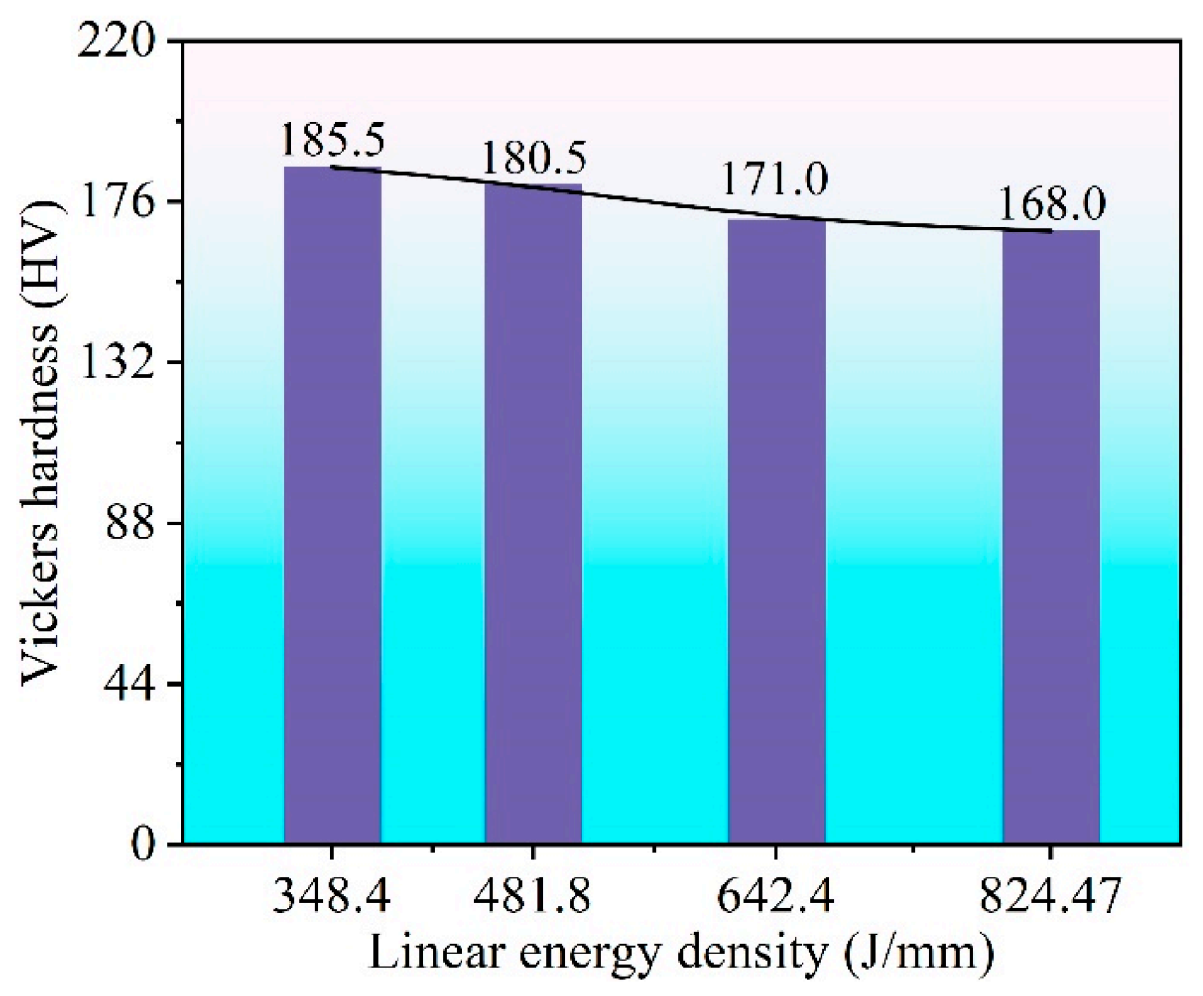
Vickers hardness of thin-wall deposits under different linear energy densities.

In addition to tensile properties, Vickers hardness tests were performed to further characterize the mechanical response of the deposited materials under different linear energy densities. As shown in Figure 11, the average Vickers hardness of the thin-wall deposits exhibits a gradual decreasing trend with increasing linear energy density. The hardness value decreases from 185.5 HV at a linear energy density of 348.40 J/mm to 180.5 HV at 481.80 J/mm, then drops to 171.0 HV at 642.40 J/mm, and finally reaches 168.0 HV at 824.47 J/mm. The overall decline is moderate, with a total reduction of approximately 9.4% across the investigated parameter range.

The decreasing hardness trend is mainly attributed to the microstructural evolution induced by elevated heat input. As discussed in the previous section, higher linear energy density raises the peak temperature and prolongs the high-temperature dwelling time in the deposition zone, which significantly promotes the growth of austenite grains. The coarsened grain structure reduces the grain boundary area per unit volume and weakens the grain boundary strengthening effect, leading to a decline in material hardness. Meanwhile, the slower cooling rate under high heat input conditions provides more sufficient time for the transformation from primary delta ferrite to austenite during solid-state cooling, resulting in a lower volume fraction of retained delta ferrite in the final microstructure. The reduction in ferrite-phase content also contributes to the decrease in overall hardness of the deposit. Furthermore, the slower cooling process under higher heat input is conducive to the release of residual stress inside the material, which also causes a certain degree of hardness reduction.

It should be noted that the variation trend of hardness differs from that of transverse tensile strength, which increases continuously with rising heat input. This discrepancy arises because transverse tensile performance is jointly dominated by inter-layer bonding quality and matrix microstructure, and the improvement in inter-layer metallurgical bonding plays a dominant role at higher heat input. In contrast, hardness mainly reflects the intrinsic deformation resistance of the deposit matrix, which is more sensitive to grain size evolution and phase-composition changes.

## 4. Conclusions

309L austenitic stainless steel thin-wall samples were successfully fabricated by cold metal transfer wire-arc additive manufacturing. The effects of process parameters, deposition paths, and linear energy density on forming characteristics, microstructure, and mechanical properties were systematically investigated. The main conclusions are as follows:

(1) Both wire-feeding speed and welding speed significantly influence the geometry and surface morphology of single-track beads. Bead width increases monotonically with wire-feeding speed, whereas reinforcement height first increases and then slightly decreases. Both bead width and reinforcement height decrease continuously with increasing welding speed. Optimal single-layer forming quality is obtained at a wire-feeding speed of 5 m/min and a welding speed of 3 mm/s.
(2) The deposition path strategy critically governs the forming quality of multi-layer deposits. The unidirectional path induces persistent heat accumulation imbalance at the component ends, causing severe end collapse, whereas the reciprocating path effectively balances inter-layer temperature distribution and suppresses end collapse. Under optimized parameters, the end collapse height remains within 5 mm after 35 layers of deposition.
(3) Linear energy density substantially regulates the as-deposited microstructure, which consists of an austenite matrix with dispersed retained delta ferrite. Increasing linear energy density promotes austenite grain growth and transforms delta ferrite from a discrete vermicular morphology to a continuous skeletal morphology, reflecting the extended duration of solid-state phase transformation during cooling.
(4) The mechanical properties exhibit pronounced anisotropy dependent on linear energy density. Longitudinal tensile strength and elongation remain stable at approximately 550 MPa and 41–45%, respectively, showing negligible linear energy density dependence. In contrast, transverse tensile strength and elongation increase continuously from 495 MPa and 33% at 348.40 J/mm to 581 MPa and 50% at 824.47 J/mm, primarily due to enhanced inter-layer metallurgical bonding that outweighs grain coarsening. The average Vickers hardness decreases gradually from 185.5 HV to 168.0 HV with increasing linear energy density.

## Figures and Tables

**Table 1:** Chemical composition of 309L stainless steel welding wire (mass fraction, %).

C	Cr	Mn	Ni	Si	S	P	Fe
0.035	24.15	1.25	13.16	0.6	0.004	0.023	Bal.

**Table 2:** Single-track single-layer deposition test scheme.

Wire-Feeding Speed (m/min)	Welding Speed (mm/s)				
	2	3	4	5	6
2	(2, 2)	(3, 2)	(4, 2)	—	—
3	(2, 3)	(3, 3)	(4, 3)	(5, 3)	—
4	(2, 4)	(3, 4)	(4, 4)	(5, 4)	(6, 4)
5	(2, 5)	(3, 5)	(4, 5)	(5, 5)	(6, 5)
6	(2, 6)	(3, 6)	(4, 6)	(5, 6)	(6, 6)

**Table 3:** Test scheme for single-track multi-layer additive manufacturing of thin-walls.

Scheme No.	Wire-Feeding Speed (m/min)	Welding Speed (mm/s)	Voltage (V)	Current (A)	Linear Energy Density (J/mm)
J-1	3	2	10.40	67	348.40
J-2	5	4	14.60	132	481.80
J-3	5	3	14.60	132	642.40
J-4	6	3	16.60	149	824.47

**Table 4:** Process parameters and cross-sectional geometric characteristics of single-track single-layer deposited beads.

Scheme No.	Welding Speed (mm/s)	Wire-Feeding Speed (m/min)	Bead Width (mm)	Reinforcement Height (mm)	Weld Reinforcement Form Factor
1	2	2	4.24	3.90	1.09
2	3	2	4.00	3.25	1.23
3	4	2	3.66	2.80	1.31
4	2	3	6.62	4.55	1.45
5	3	3	5.60	3.75	1.49
6	4	3	5.12	3.00	1.71
7	5	3	4.82	2.85	1.69
8	2	4	7.94	4.90	1.62
9	3	4	6.48	4.10	1.58
10	4	4	5.80	3.70	1.57
11	5	4	5.50	3.05	1.80
12	6	4	5.40	2.85	1.89
13	2	5	10.40	4.90	2.12
14	3	5	8.72	4.00	2.18
15	4	5	7.76	3.60	2.16
16	5	5	7.54	3.20	2.36
17	6	5	7.10	2.45	2.90
18	2	6	12.00	4.80	2.50
19	3	6	10.20	4.00	2.55
20	4	6	9.30	3.75	2.48
21	5	6	8.40	3.80	2.21
22	6	6	8.30	2.90	2.86

## Data Availability

The original contributions presented in this study are included in the article. Further inquiries can be directed to the corresponding author.
